# Field rates of Sivanto^™^ (flupyradifurone) and Transform^®^ (sulfoxaflor) increase oxidative stress and induce apoptosis in honey bees (*Apis mellifera* L.)

**DOI:** 10.1371/journal.pone.0233033

**Published:** 2020-05-21

**Authors:** Priyadarshini Chakrabarti, Emily A. Carlson, Hannah M. Lucas, Andony P. Melathopoulos, Ramesh R. Sagili

**Affiliations:** Department of Horticulture, Oregon State University, Corvallis, Oregon, United States of America; University of North Carolina at Greensboro, UNITED STATES

## Abstract

Pesticide exposures can have detrimental impacts on bee pollinators, ranging from immediate mortality to sub-lethal impacts. Flupyradifurone is the active ingredient in Sivanto^™^ and sulfoxaflor is the active ingredient in Transform^®^. They are both relatively new insecticides developed with an intent to reduce negative effects on bees, when applied to bee-attractive crops. With the growing concern regarding pollinator health and pollinator declines, it is important to have a better understanding of any potential negative impacts, especially sub-lethal, of these pesticides on bees. This study reports novel findings regarding physiological stress experienced by bees exposed to field application rates of these two insecticides via a Potter Tower sprayer. Two contact exposure experiments were conducted—a shorter 6-hour study and a longer 10-day study. Honey bee mortality, sugar syrup and water consumption, and physiological responses (oxidative stress and apoptotic protein assays) were assessed in bees exposed to Sivanto^™^ and Transform^®^, and compared to bees in control group. For the longer, 10-day contact exposure experiment, only the Sivanto^™^ group was compared to the control group, as high mortality recorded in the sulfoxaflor treatment group during the shorter contact exposure experiment, made the latter group unfeasible to test in the longer 10-days experiment. In both the studies, sugar syrup and water consumptions were significantly different between treatment groups and controls. The highest mortality was observed in Transform^®^ exposed bees, followed by the Sivanto^™^ exposed bees. Estimates of reactive oxygen/nitrogen species indicated significantly elevated oxidative stress in both pesticide treatment groups, when compared to controls. Caspase-3 protein assays, an indicator of onset of apoptosis, was also significantly higher in the pesticide treatment groups. These differences were largely driven by post exposure duration, indicating sub-lethal impacts. Further, our findings also emphasize the need to revisit contact exposure impacts of Sivanto^™^, given the sub-lethal impacts and mortality observed in our long-term (10-day) contact exposure experiment.

## Introduction

Pesticides can negatively impact managed bee stocks and native bee populations [1–8]. These impacts include lethal effects, as well as a host of sub-lethal effects, including cognitive impairments [6,7,9–13], flight and homing disruptions [3, 14,15] and detrimental physiological alterations [16–19]. Although the underlying physiological mechanisms that cause direct lethality of insecticides to bees are well-resolved, the connection between physiological processes and sub-lethal effects are not well understood. The lethal toxicity is currently captured in pesticide risk assessments, but recent research has demonstrated a need to better understand the sub-lethal impacts of pesticides along with the physiological underpinnings of such impacts.

Researchers have observed that pesticides can disrupt physiological processes that are unrelated to the intended modes of action. Pesticide exposure, for example, can result in oxidative stress [16,20] and apoptosis [18,21] in bees, which is different than the intended modes of action such as disrupting neurological functions (organophosphates and neonicotinoids) or axonic membrane functions (synthetic pyrethroids). If the production of reactive oxygen (ROS) and nitrogen (RNS) species exceeds the body’s natural anti-oxidative defense mechanisms [22,23], then oxidative stress occurs in a number of organisms, including honey bees [24]. An imbalance of excess ROS/RNS-producing environmental factors [25] results in oxidative damage to the organisms. Severe physiological impairments and damage to important macromolecular structures may arise, thereby disrupting vital biological processes [26,27]. Increased systemic oxidative stress, generated by higher levels of ROS/RNS, can play an important role in pesticide toxicity [28–30]. High ROS/RNS levels are also indicative of an onset of apoptosis [30–32]. Apoptosis is a form of programmed cell death [33]. The sublethal impacts of pesticide-induced apoptosis in bees is a serious concern, with effects ranging from midgut cellular lesions to neuronal degeneration in brains [18,33,34]. Caspase-3, a crucial protein in the apoptotic pathway in all organisms, is considered an important marker of apoptotic onset [35,36]. Increased levels of both ROS and caspase-3 are thus indicative of physiological stress. The negative impacts of oxidative stress and apoptosis on individual bees may have cascading effects on the entire colony. For example: oxidative stress may induce precocious foraging (rapid behavioral maturation) leading to colony decline [37].

Our study focuses on the physiological effects of field application rates of two new insecticides that have been developed with an intent to reduce negative effects on bees, when applied to bee-attractive crops. Flupyradifurone, the active ingredient in Sivanto^™^ (Bayer Crop Science, Germany), and sulfoxaflor, the active ingredient in Transform^®^ (Corteva Agriscience, United States), are two insecticides recently registered for use in the United States [38,39] and are developed to be more compatible with bee health. While Sivanto^™^ has no restrictions for application during bloom, Transform^®^ has a relatively short residual time on petals and leaf-material and can be legally used during bloom “when managed bees and native pollinators are least active, e.g., 2 hours prior to sunset or when the temperature is below 50°F at the site of application” [40]. Both active ingredients are systemic, and act as agonists of the nicotinic acetylcholine receptor (nAChR). While both products are considered acutely toxic at field-applied rates when consumed by bees, Sivanto^™^ is different in that the US labels indicate the product is “not toxic to bees through contact exposure”.

The present study investigated if field application rates of Sivanto^™^ and Transform^®^ result in sub-lethal physiological effects on honey bees, which extend beyond the recognized mode-of-action of these products. Understanding the lethal and sublethal impacts of Sivanto^™^, upon contact exposures, is particularly important since its label indicates that the product lacks toxicity through contact exposures. We investigated these questions in laboratory cage experiments, where newly emerged adult honey bees were directly sprayed with field application rates recommended on the product labels of Transform^®^ and Sivanto^™^. We examined survival at 6 hours (lethal effects) and 10 days (sub-lethal effects) in two separate contact exposure experiments. Since pesticide exposures also have the potential to alter food consumption in treated bees [19,41] and affect carbohydrate metabolism, we also measured sugar syrup consumptions. Additionally, physiological impacts were assessed by estimating both oxidative stress (ROS/RNS assay) and apoptosis (caspase-3 protein activity) in the treatment groups.

## Methods

### Sampling of honey bees for the study

All experiments were conducted in May 2019. The honey bees for the experiments were obtained from six colonies (headed by open mated sister-queens to ensure genetic similarities) at the Oregon State University apiaries in Corvallis, Oregon, USA. The sister-queens were purchased from Jackie Park-Burris Inc. (CA, USA). All six colonies were strong and healthy with low *Varroa* levels and no visible symptoms of any brood disease. Three frames of capped brood from each colony (18 frames in total) were placed in cardboard nucleus hive boxes and placed in an incubator (Percival Intellus I-36VL, Percival Scientific Inc., USA) overnight at 33.5°C and 55% RH, allowing bees to emerge. The following day, newly emerged bees from all frames were pooled and gently hand-mixed to ensure a homogenized population. Then, 150 bees were randomly allocated to each replicate cage of each treatment group (see below). Cylindrical cages, constructed with 0.3175cm hardware cloth and approximately 25.4cm tall and 12.7cm in diameter, were used for this study [42]. Water and 40% sugar syrup (w/v) were provided to bees *ad libitum* via inverted glass vials on the top of all cages. Evaporation control cages (cages with only sugar syrup and water, and no honey bees) were also included in the study to account for the effects of evaporation. We conducted two separate contact exposure experiments (see below); honey bees for both these experiments were collected and housed as described above.

### Experimental design and pesticide exposures

Each of the three experimental groups—control (no pesticide exposure), Sivanto^™^ (flupyradifurone) and Transform^®^ (sulfoxaflor)—contained four replicate cages. Sivanto^™^ Prime (Bayer, active ingredient: Flupyradifurone, 17.09%) and Transform^®^ WG (Corteva Agriscience, USA, active ingredient: Sulfoxaflor, 50%) were purchased commercially. Each insecticide was mixed at field application rates: (a) Flupyradifurone: 218 μl Sivanto^™^ Prime in 20 ml water (Sivanto^™^ Prime package label: 14 fl oz Ac^-1^ having spray volume 10 gallons Ac^-1^) and (b) Sulfoxaflor: 33.702 mg Transform^®^ WG in 20 ml water (Transform^®^ WG package label: 2.25 oz Ac^-1^ having spray volume 10 gallons Ac^-1^). The concentrations of Sivanto^™^ used in this study represent recommended high rates (in the rate range 7 to 14 fl oz Ac^-1^) in crops such as in citrus fruits and apples to manage pests such as aphids and psyllids (Sivanto^™^ Prime label). Similarly, the concentrations of Transform^®^ used in our study represent recommended high rates (in the rate range 0.75 to 2.75 oz Ac^-1^) for controlling aphids, psyllids and whiteflies in crops such as citrus, apples and other fruiting vegetable crops (Transform^®^ WG label).

To expose experimental bees to the pesticides, all 150 bees in a given cage were immobilized with carbon dioxide (30 second exposure at 15psi; AirGas, Albany OR) and spread out in a single, even layer on a 140-mm plastic petri dish. Honey bees received a single dose of pesticide via a Potter Spray Tower (Burkard Manufacturing Co. Ltd., England) [43,44]. Each Petri dish of anesthetized bees was placed on the Potter spray tower stage and sprayed with 2 ml of the appropriate insecticide solution or water (for cages in the control group), using an air pressure of 47 kPa (6.8 psi) and a spray distance of 22 cm [44,45]. The bees were then transferred to a clean Petri dish and placed back in their designated cage. The Potter sprayer delivered the pesticide solution as a fine mist, which formed a uniform layer on the bees, without creating any visible droplets.

Two contact exposure experiments were conducted: one for 6 hours, and another for 10 days. The short-term 6-hours experiment was initially conducted to understand the lethal toxicity of both pesticides to honey bees. Survival and physiological responses were compared between the treatment groups. Since a majority of the honey bees in the Transform^®^-exposed group died within six hours in the shorter contact exposure experiment, the Transform^®^ group was excluded from the second, longer 10-day contact exposure experiment. The second experiment was conducted to assess the lethal (mortality observed at the endpoint of the experiment) and the sublethal impacts (physiological alterations observed in the surviving honey bees) of acute contact exposure of Sivanto^™^ on honey bee physiology. A control group (bees sprayed with only water), was included in both (6-hours and 10-day) experiments.

### Survival analyses

Honey bee mortality in each cage was recorded every hour for the 6-hour experiment and daily for the 10-day experiment. Total mortality was calculated at the end of the experiment for each replicate cage in all treatment and control groups. Kaplan-Meier Log Rank Survival analysis was performed [16,46,47] using GraphPad Prism Version 8.0.2 (GraphPad Software, San Diego, USA). Survival curves were calculated for a period up to 6 hours for the shorter contact exposure experiment and 10 days for the longer contact exposure experiment.

### Consumption analyses

In the 6-hour experiment, water and sugar syrup consumption were recorded at the end of the experiment. Each glass vial was initially filled with 30 ml of sugar syrup or water and the volume of liquid remaining in each vial was recorded at the end of the experiment, and subtracted from the initial volume given (30 ml). The volume of liquid lost through evaporation (from vials in evaporation control cages) was subtracted from the volume lost in vials from honey bee-containing cages. This was then adjusted to the number of live honey bees at the end of the experiment to calculate consumption per bee. Similar estimations for water and sugar syrup consumptions were made each day for the 10-day experiment, where water and sugar syrups were replaced daily.

At the end of both the 6-hours and 10-days experiments, five live honey bees were sampled from each replicate cage in the Sivanto^™^ and control groups for estimating oxidative stress. Five live honey bees were also collected from those cages for estimating caspase-3 activity. Likewise, we sampled five live honey bees for each assay (10 live bees in total) from each of the Transform^®^-treatment group replicate cages at the end of the 6-hour experiment. All the sampled bees were immediately frozen at -80 °C until time of analysis.

### Activity assays of reactive oxygen species (an indicator of pesticide induced oxidative stress)

As mentioned before, five honey bees from each replicate cage, for every treatment group, were pooled together. Whole honey bees were homogenized in phosphate buffered saline (PBS, Sigma Aldrich, USA; 10 mM phosphate, 2.7 mM potassium chloride and 137 mM sodium chloride, pH 7.4) using a Precellys 24 tissue homogenizer (Bertin Instruments, Rockville, USA) at 6000 rpm for three cycles of 30 seconds per cycle. The tissue homogenates were then centrifuged at 10,000g for 5 minutes at 4°C (Eppendorf model 5430R, Eppendorf, USA) and supernatants were immediately assayed for reactive oxygen/nitrogen species as described below.

Oxidative stress was measured by assaying total reactive oxygen species (ROS) and total reactive nitrogen species (RNS) activities using OxiSelect^™^ In vitro ROS/RNS Assay Kit—Green Fluorescence (Cell Biolabs. Inc., San Diego, USA) [48,49] using the manufacturer’s microplate assay protocols. Briefly, the assay principle is based on oxidizing a fluorogenic probe (2’,7’-dichlorodihydrofluorescin DiOxyQ) to a fluorescent product (2’,7’-dichlorodihydrofluorescein) in the presence of ROS and RNS. The fluorescence intensity is proportional to the amount of ROS/RNS present in the sample. Fluorescence was measured at 480 nm excitation and 530 nm emission wavelengths by a microplate reader (Biotek Synergy 2, BioTek Instruments, USA) using a hydrogen peroxide standard curve. The pooled tissue homogenates from each replicate cage were assayed in duplicate in a 96-well plate. We used general protein content to normalize the results of oxidative stress assays across all samples, such that total ROS/RNS activity is expressed as μmol μL^-1^ μg^-1^ protein. Therefore, protein estimations of the homogenate supernatants were performed using a BCA Assay Kit (Thermo Scientific, USA) following the microplate assay protocol, and the absorbance was measured at 562 nm (BioTek Synergy 2 plate reader, BioTek Instruments, USA) [42].

### Caspase-3 protein activity assay (an indicator of cell death/apoptosis)

For estimating caspase-3 activity, five honey bees were sampled and pooled from each of the experimental cages (for all treatments and controls) and homogenized and centrifuged as described above for ROS/RNS assays. The resulting supernatants were immediately assayed. Apoptosis was estimated by detecting the increase in caspase-3 activity using the fluorometric caspase-3 assay (Abcam, Cambridge, USA) following manufacturer’s protocols [50,51]. Sample supernatants were assayed in duplicate and fluorescence was measured at 400 nm excitation and 505 nm emission by a microplate reader (Biotek Synergy 2, BioTek Instruments, USA). As with ROS/RNS assays, caspase-3 activity was normalized using general protein content. Thus, protein content was quantified using a BCA Assay Kit (Thermo Scientific, USA) as described above [42]. Caspase-3 activity is expressed as U μg^-1^ protein.

### Statistical analyses

All statistical analyses were performed using GraphPad Prism Version 8.0.2 (GraphPad Software, San Diego, USA). Shapiro-Wilk test for normality was conducted for every dataset. For comparing two groups, two tailed t-tests were performed for data that were normally distributed and Mann Whitney U test for non-parametric tests. For parametric tests, multiple comparisons were performed using One-Way ANOVA with Tukey’s Posy Hoc comparisons. Kruskal-Wallis test with Dunn’s multiple comparisons were performed for data that were not normally distributed. The data for caspase-3 was log transformed. Means are presented as means ± standard errors of means.

## Results

### Survival

#### Six-hour contact exposure experiment

**Fig 1:**
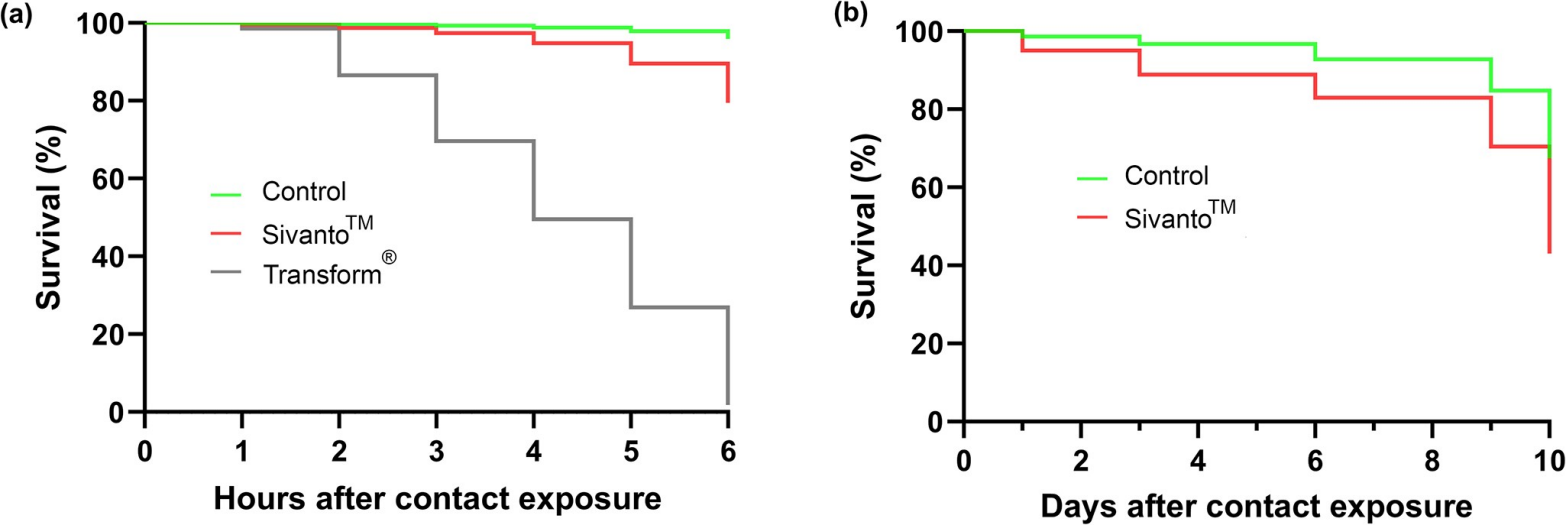
Honey bee survival during the (a) 6-hour and (b) 10-day contact exposure experiments when exposed to Transform^®^ (active ingredient sulfoxaflor) and Sivanto^™^ (active ingredient flupyradifurone). The control group was only sprayed with water in the Potter Tower.

The survival curves were significantly different among the experimental groups in the 6-hours contact exposure experiment (Kaplan Meier, Log Rank test χ2 = 4719, df = 2 and p < 0.001) with the highest bee survival recorded in the control group, followed by the Sivanto^™^ and Transform^®^ groups (Fig 1a).

#### Ten-day contact exposure experiment

Log-rank test indicated significant differences in bee survival among experimental groups (Kaplan Meier, Log Rank test χ2 = 192.6, df = 1 and p < 0.001). While more bees survived in the control group compared to the Sivanto^™^ group, this difference became pronounced by the end of the experiment when control cages had 70% survival compared to 45% in the Sivanto^™^ group (Fig 1b).

### Consumption of water and sugar syrup

#### Six-hour contact exposure experiment

**Fig 2:**
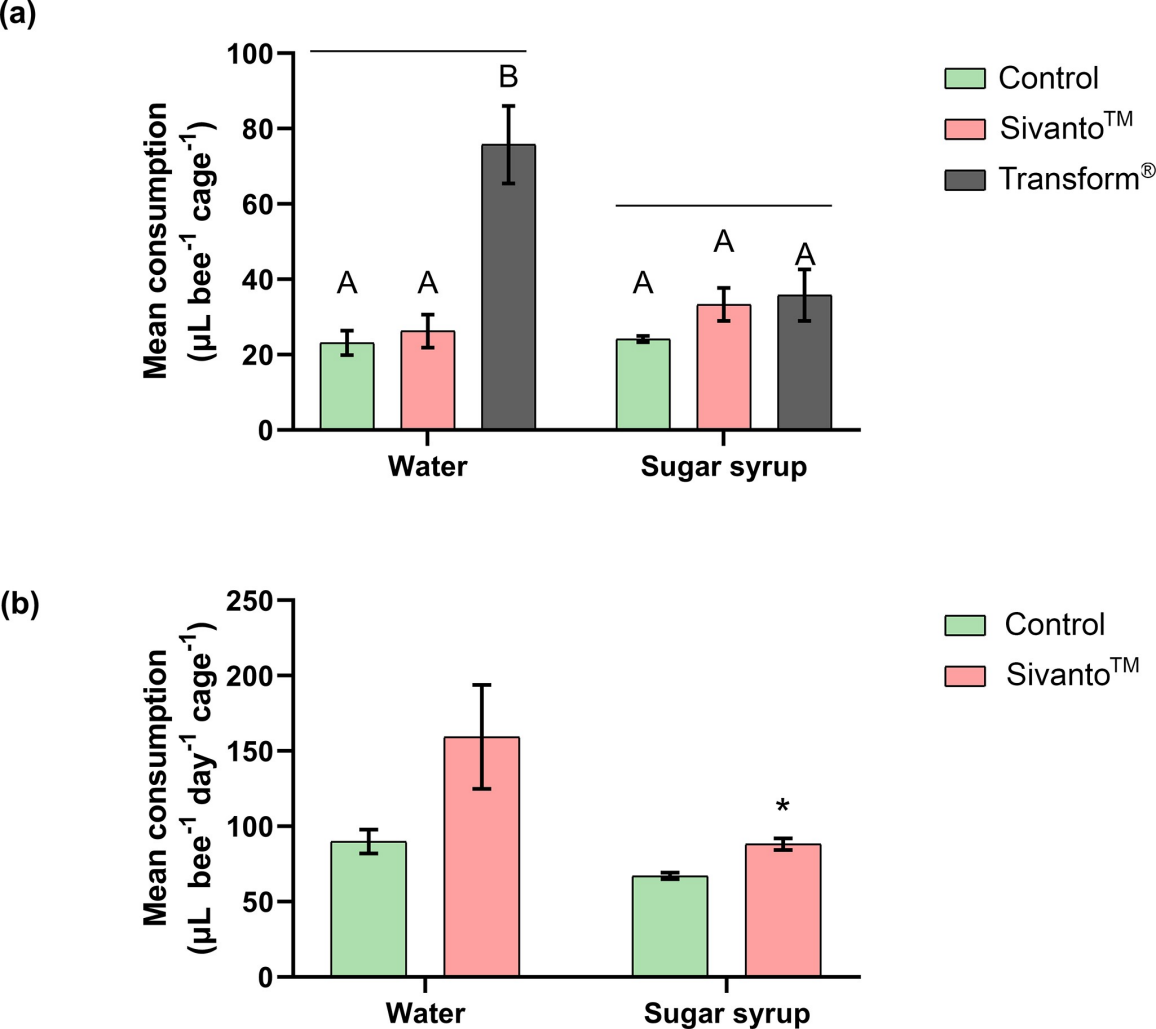
Mean water and sugar syrup consumptions during the (a) 6-hour and (b) 10-day contact exposure experiments when treatment group of honey bees were exposed to Transform^®^ (active ingredient sulfoxaflor) and Sivanto^™^ (active ingredient flupyradifurone). The control group was only sprayed with water in the Potter Tower. Error bars indicate SEM. Different alphabets indicate statistical significance at p < 0.05, as analyzed by Tukey’s Post Hoc tests. * indicates statistical significance at p < 0.05.

Consumption of water in the 6-hour experiment was significantly different between the treatment groups (One-way ANOVA with Tukey’s Post Hoc comparisons, F_(2,9)_ = 19.28, p < 0.001) (Fig 2a). Control, Sivanto^™^ and Transform^®^ treatment groups consumed 23.14±3.23, 26.25±4.39 and 75.78±10.29 μL bee^-1^ cage^-1^ respectively. However, sugar syrup consumption was not significantly different (One-way ANOVA with Tukey’s Post Hoc comparisons, F_(2,9)_ = 1.706, p = 0.2354) with control bees consuming 24.14±0.84 μL bee^-1^ cage^-1^, Sivanto^™^-exposed bees consuming 33.32±4.39 μL bee^-1^ cage^-1^ and Transform^®^-exposed bees consuming 35.79±6.80 μL bee^-1^ cage^-1^ of sugar syrup (Fig 2a).

#### Ten-day contact exposure experiment

For the 10-day experiment, water consumption was not significantly different between the experimental groups (t = 1.962, df = 6, p = 0.0974), even though higher consumption was observed in the Sivanto^™^ group (159.32±34.43 μL bee^-1^ day^-1^ cage^-1^), when compared with the control group (89.98±7.90 μL bee^-1^ day^-1^ cage^-1^) (Fig 2b). Sugar syrup consumption differed significantly between experimental groups (t = 4.766, df = 6, p < 0.05), with control and Sivanto^™^ groups consuming sugar syrup as follows: 73.36±2.16 μL bee^-1^ day^-1^ cage^-1^ and 81.84±3.86 μL bee^-1^ day^-1^ cage^-1^ respectively (Fig 2b).

### Pesticide induced oxidative stress in honey bees

#### Six-hour contact exposure experiment

**Fig 3:**
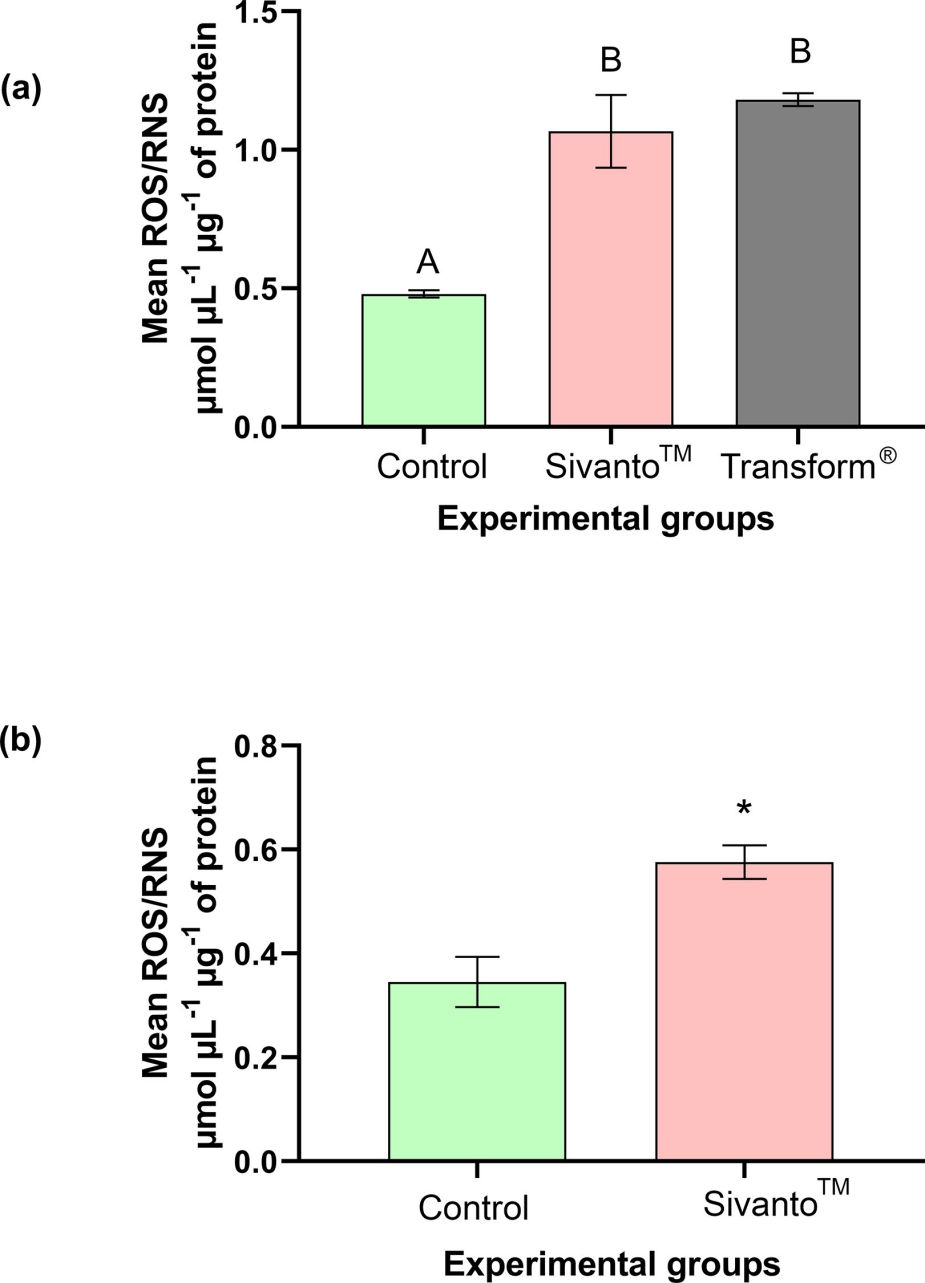
Mean ROS/RNS concentration in bees that were exposed (contact exposure) to Transform^®^ (active ingredient sulfoxaflor), Sivanto^™^ (active ingredient flupyradifurone) and water (Control) in the (a) 6-hour and (b) 10-day contact exposure experiments. Error bars indicate SEM. Different alphabets indicate statistical significance at p < 0.05, as analyzed by Tukey’s Post Hoc tests. * indicates statistical significance at p < 0.05.

The total ROS/RNS activity was significantly different between the treatment groups (One-way ANOVA with Tukey’s Post Hoc comparisons, F_(2,21)_ = 23.56, p < 0.001) (Fig 3a). Both the Sivanto^™^ exposed honey bees and the Transform^®^ exposed honey bees expressed significantly higher ROS/RNS activities, when compared with the control group (2.19±0.24 and 2.47±0.05 fold increases respectively). There was no significant difference in ROS/RNS activity between Sivanto^™^ and Transform^®^ treatment groups (p = 0.5615). The mean ROS/RNS activity values for control, Sivanto^™^ and Transform^®^ treatment groups were 0.48±0.01 μmol μL^-1^ μg^-1^ protein, 1.07±0.13 μmol μL^-1^ μg^-1^ protein^and^ 1.18±0.023 μmol μL^-1^ μg^-1^ protein respectively (Fig 3a).

#### Ten-day contact exposure experiment

Total ROS/RNS activity significantly differed between the treatment groups (Mann Whitney U = 4, p < 0.05) (Fig 3b). The total ROS/RNS activity was significantly higher (1.975±0.33 fold increase) in the Sivanto^™^ treatment group when compared to the control. The mean ROS/RNS activities were 0.35±0.05 μmol μL^-1^ μg^-1^ protein and 0.58±0.03 μmol μL^-1^ μg^-1^ protein in control and Sivanto^™^ groups respectively (Fig 3b).

### Pesticide induced cell death (apoptosis) in honey bees

#### Six-hour contact exposure experiment

**Fig 4:**
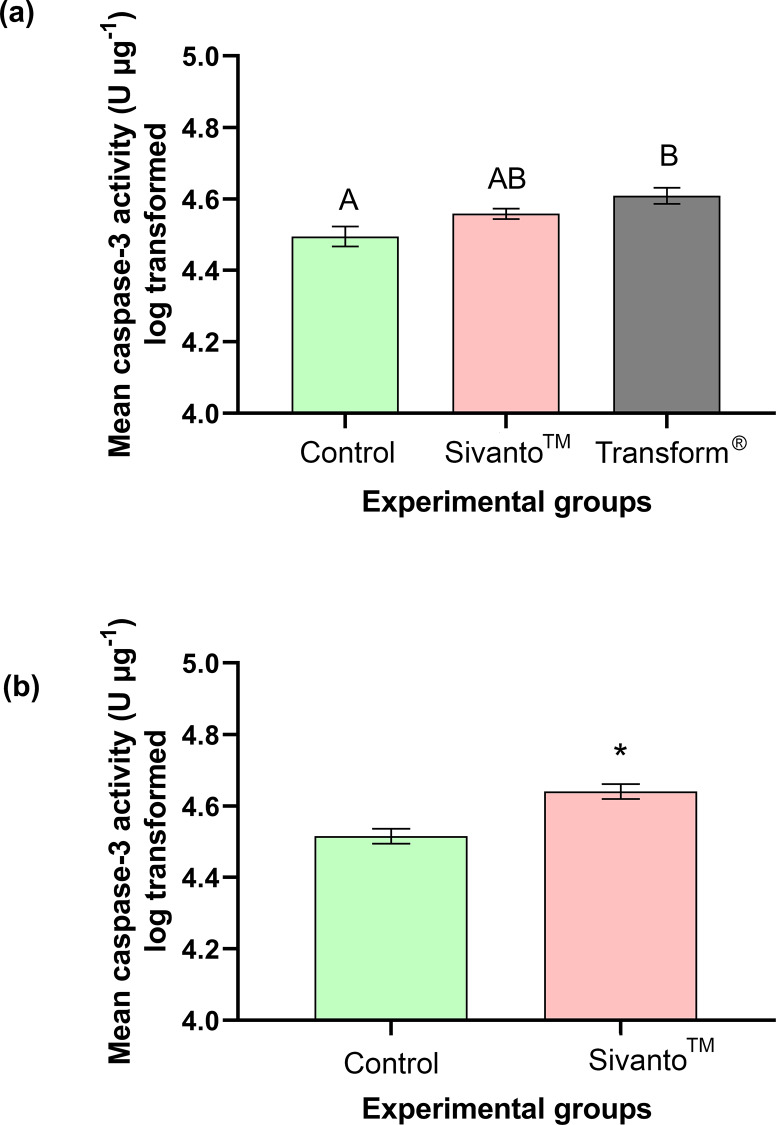
Mean caspase-3 activity in bees exposed to Transform^®^ (active ingredient sulfoxaflor), Sivanto^™^ (active ingredient flupyradifurone) and water (Control) in the (a) 6-hours and (b) 10-days contact exposure studies. Error bars indicate SEM. Different alphabets indicate statistical significance at p < 0.05, as analyzed by Tukey’s Post Hoc tests. * indicates statistical significance at p < 0.05.

The caspase-3 activity was significantly different between treatment groups (Kruskal-Wallis test H = 12.26, p < 0.05) (Fig 4a). The bees in the Transform^®^ treatment group had significantly higher caspase-3 activity (1.31±0.09 fold higher) when compared to bees in the control group (Dunn’s multiple comparisons, z = 3.5, p < 0.05) (Fig 4a). No significant differences were observed in caspase-3 activities between Sivanto^™^ exposed bees and bees in the control group (Dunn’s multiple comparisons, z = 1.803, p = 0.2141). Similarly, the caspase-3 activity was not significantly different between Sivanto^™^ and Transform^®^ exposed bees (Dunn’s multiple comparisons, z = 1.697, p = 0.2691). The mean caspase-3 activity for the control, Sivanto^™^ and Transform^®^ treatment groups were 3167.55±0.03 U μg^-1^ protein, 3633.26±0.01 U μg^-1^ protein and 4105.023±0.02 U μg^-1^ protein respectively (Fig 4a).

#### Ten-day contact exposure experiment

Caspase-3 activity was significantly different between treatment groups (t = 4.241, df = 14, p < 0.001) (Fig 4b). Caspase-3 activity was 1.35±0.09 times higher in the Sivanto^™^-exposed group than in the control group (Fig 4b). Mean caspase-3 activities were 3302.83±0.02 U μg^-1^ protein and 4406.45±0.02 U μg^-1^ protein in control and Sivanto^™^ groups respectively (Fig 4b).

## Discussion

This study reports, for the first time, increased oxidative stress and onset of apoptosis in honey bees exposed to two relatively recently registered insecticides—Transform^®^ and Sivanto^™^. Our findings provide new insights on sub-lethal and lethal impacts of contact exposures of field application rates of these two pesticides (formulated products) on honey bees. Both bee mortality and parameters related to physiological stress in honey bees were measured for a comprehensive understanding of potential negative impacts of these pesticides.

A majority of the honey bees exposed to Transform^®^ died within the six hours after initiation of the experiment, which confirms severe toxicity of Transform^®^ to bees when exposed directly to field application rates recommended on the label. Further, our findings also confirm that Sivanto^™^ is not directly lethal to honey bees following contact exposure. Our 10-day survival results, however, revealed evidence that field-rates of Sivanto^™^ may reduce adult survival. This suggests that even though Sivanto^™^ is apparently less toxic than Transform^®^, it may reduce honey bee longevity. This is further evident in the survival analyses, where honey bees in the control groups exhibited the highest survival in both contact exposure studies. Consequently, we provide data that contact exposure routes, in addition to known oral routes of exposure, may be an additional source of acute toxicity of Sivanto^™^. Notably, our work investigated the direct application of Sivanto^™^ to bees, not the contact of bees with field-weathered residues of the formulated product on leaves and flower petals. Future studies should investigate whether similar effects can be replicated when bees contact residues found on plants following treatments.

Consumption of sugar syrup was not significantly different in the 6-hour experiment. However, Sivanto^™^ exposed honey bees consumed significantly more sugar syrup than the control honey bees over the 10-day period. Pesticide exposures are known to alter sugar syrup consumption in treated bees [19,41]. The energetics of carbohydrate metabolism and regulation may be responsible for such consumption differences. Pesticides are reported to impact glucose and glycogen levels in bee tissues, altering various carbohydrate signaling and molecular pathways [19]. Examining these mechanisms was beyond the scope of this study. The differential syrup consumption we observed, however, further indicates that pesticides may influence bee metabolic function. For similar metabolic reasons, water consumption may also have differed between the treatment groups in both contact exposure studies.

The present study further illustrates pesticide induced oxidative stress in honey bees. In the shorter 6-hour experiment, Transform^®^ exposed honey bees exhibited the highest oxidative stress (significantly higher than control) when compared with honey bees in other treatment groups. In both Sivanto^™^ contact groups, ROS/RNS was significantly higher than the control group. However, it should be noted that mean ROS/RNS concentrations were less at the end of the longer 10-day experiment, when compared with the shorter 6-hour exposure experiment. This may indicate an immediate oxidative stress in the Sivanto^™^ groups during the shorter study, which may have been physiologically mitigated over the 10-day duration as seen in the longer contact exposure study. Even though *Apis mellifera* has fewer detoxification genes compared to other insects [52], they are known to elevate antioxidant enzymes (for example: catalase, superoxide dismutase, xanthine oxidase etc.) [16] or develop tolerance to mitigate such physiological stressors in their bodies to offset pesticide induced oxidative stress [53]. Both flupyradifurone and sulfoxaflor target insect nAChR and other molecular targets may be potentially affected as well [54]. A number of neonicotinoids are known to increase production of reactive oxygen species in honey bees, but direct evidence of increased oxidative stress in bees, upon contact exposures to Sivanto^™^ (active ingredient flupyradifurone) and Transform^®^ (active ingredient sulfoxaflor) at field-application rates, was lacking [54].

Initiation of cell death and apoptosis was also evident in both contact exposure experiments. Caspase-3 is an important marker of apoptosis, and has been reported to increase under pesticide stress in all organisms, including honey bees [30,33,35,36]. Significantly increased activity of caspase-3 in Transform^®^-treated honey bees in the 6-hour contact exposure experiment (compared to control), indicated physiological damage that may occur from this pesticide at field application rates. In bees exposed to Sivanto^™^, caspase-3 activity was significantly elevated (compared to control) at the end of the 10-day period (longer study), and not after 6 hours (short duration study), indicating the sublethal effects of this pesticide on bees in the long term. We speculate that apoptosis was potentially initiated in the bees that survived until the end of the experiment, but was not discerned during the short term (after six hours). This finding is important when assessing impacts of such pesticides in the field over a longer duration of time.

Physiological impacts of pesticides, for example oxidative stress and apoptosis, can render individual honey bees incapable of performing their tasks smoothly, thereby affecting the colony performance as well. Previous studies have shown that different species of honey bees exhibit different anti-oxidant enzyme responses to field-realistic oral exposures pesticides (*Apis cerana* and *Apis dorsata*) [16], as well as varying conditions of temperature and humidity (*Apis cerana* and *Apis mellifera*) [55]. Further, pesticide-induced oxidative stress, coupled with other stressors, can reduce longevity in honey bees [56]. Thus, reduced longevity resulting from oxidative stress could negatively affect colony population and ultimately compromise colony fitness. Previous studies have also asserted the need to conduct similar long-term monitoring for pesticide exposures [57,58], which may help assess the long-term impacts of such exposures on colony health. Even though the present study uses total ROS/RNS and caspase-3 activity assays as indicators for physiological stress, future studies should look into other proteins of the apoptotic pathways and antioxidant enzymes.

Recent studies on flupyradifurone [8,54,59] and sulfoxaflor [60,61] have demonstrated lethal and sublethal impacts of these pesticides in terms of survival, cognition and abnormal behavior in honey bees. However, to our knowledge, the physiological underpinnings of these impacts due to direct contact exposures are not known. Our study has attempted to address this gap in knowledge. Further, our findings also suggest the need to revisit the potential impacts of contact exposure of Sivanto^™^;, given the sub-lethal impacts and reduced longevity in honey bees observed in our long-term contact exposure experiment (10-day experiment). Future research should focus on the impacts of such field-relevant contact exposures across multiple population cohorts at different seasonal time points. With the recent Environmental Protection Agency approval for use of both flupyradifurone and sulfoxaflor, and with the growing concern regarding pollinator health, it is important to better understand any potential negative impacts (especially sub-lethal) of these pesticides on bees.

## Supporting information

S1 Data (XLSX)
